# Mechanism for generation of left isomerism in *Ccdc40* mutant embryos

**DOI:** 10.1371/journal.pone.0171180

**Published:** 2017-02-09

**Authors:** Kelsey F. Sugrue, Irene E. Zohn

**Affiliations:** 1 Institute for Biomedical Sciences, The George Washington University, Washington, DC, United States of America; 2 Center for Neuroscience Research, Children's Research Institute, Children’s National Medical Center, Washington, DC, United States of America; University of Colorado Boulder, UNITED STATES

## Abstract

Leftward fluid flow in the mouse node is generated by cilia and is critical for initiating asymmetry of the left-right axis. *Coiled-coil domain containing-40* (*Ccdc40*) plays an evolutionarily conserved role in the assembly of motile cilia and establishment of the left-right axis. Approximately one-third of *Ccdc40*^*lnks*^ mutant embryos display situs defects and here we investigate the underlying mechanism. *Ccdc40*^*lnks*^ mutants show delayed induction of markers of the left-lateral plate mesoderm (L-LPM) including *Lefty1*, *Lefty2* and *Nodal*. Consistent with defective cilia motility compromising fluid flow across the node, initiation of asymmetric perinodal *Cerberus like-2* (*Cerl2*) expression is delayed and then randomized. This is followed by delayed and then randomized asymmetric *Nodal* expression around the node. We propose a model to explain how left isomerism arises in a proportion of *Ccdc40*^*lnks*^ mutants. We postulate that with defective motile cilia, *Cerl2* expression remains symmetric and Nodal is antagonized equally on both sides of the node. This effectively reduces Nodal activation bilaterally, leading to reduced and delayed activation of Nodal and its antagonists in the LPM. This model is further supported by the failure to establish *Nodal* expression in the left-LPM with reduced Nodal gene dosage in *Ccdc40*^*lnks/lnks*^;*Nodal*^*LacZ/+*^ mutants causing a predominance of right not left isomerism. Together these results suggest a model where cilia generated fluid flow in the node functions to ensure robust Nodal activation and a timely left-sided developmental program in the LPM.

## Introduction

Vertebrates have a conserved asymmetric arrangement of visceral organs along the left-right body axis. Organs such as the heart, liver, spleen, stomach and intestine form in the midline and become asymmetrically positioned during their morphogenesis, whereas, other structures such as the lungs acquire distinct right versus left morphologies. Failure to properly specify this axis results in laterality defects where the asymmetric organization of the viscera is altered [1–3]. Heterotaxia syndromes include right isomerism, left isomerism and other discordant asymmetries of the viscera, whereas the complete reversal of the left-right axis results in situs inversus [4–6]. In humans, laterality defects occur in approximately 1 out of every 10,000 live births resulting in defects in development of the gastrointestinal tract, spleen and heart [1, 7, 8].

In the mouse, the first known left-right asymmetry occurs around the node during late gastrula stages where overlapping positive and negative feedback loops amplify initial asymmetries in gene expression. Initially, *Nodal*, *Wnt* and their antagonist *Cerberus like-2* (*Cerl2*) are symmetrically expressed in perinodal crown cells [9–12]. Initiation of leftward fluid flow across the node results in increased intracellular calcium in perinodal crown cells on the left and triggers degradation of *Cerl2* mRNA [13, 14]. Decreased *Cerl2* releases inhibition of Wnt and Nodal [12, 14]. Subsequently, Wnt signaling on the left further inhibits *Cerl2* expression and Nodal positively regulates its own expression amplifying initial asymmetries [12, 14, 15]. Other signaling pathways also participate in regulation of this gene network including Notch, which is required for the initial induction of *Nodal*, is regulated by Wnt, involved in ciliogenesis and utilizes cilia for signaling [16–21].

Asymmetric perinodal Nodal signaling is transmitted to the lateral plate mesoderm (LPM) [9, 22–24]. In the left-LPM (L-LPM), Nodal positively regulates its own expression and induces expression of its antagonists *Lefty1* and *Lefty2* to limit Nodal activity [9, 15, 22–28]. *Lefty1* expression is also induced in the midline creating a barrier to the transfer of Nodal to the right LPM (R-LPM) [27, 29]. During subsequent development, these initial asymmetries are stabilized and propagated to direct asymmetric growth of the visceral organs. Bilateral *Nodal* expression in the LPM results in the formation of two left sides or left isomerism [29–31]. By contrast, when Nodal signaling is deficient, *Nodal* and *Lefty1/2* expression is not established in the LPM on either side leading to specification of two right sides or right isomerism [9, 24, 32].

The initial bias in perinodal *Cerl2* expression depends on the leftward fluid flow generated by movement of cilia in the node [14]. The first indication that cilia were important for specifying the orientation of the left-right axis came from the observation that laterality defects were often associated with Kartagener Syndrome (later renamed Primary Ciliary Dyskinesia—PCD) characterized by chronic bronchiectasis, rhinitis, sinusitis and otitis media [33, 34]. Identification and study of numerous mutant mouse models with defects in cilia formation and/or movement and left-right axis formation provide compelling support for the idea that cilia are important for generation of leftward fluid flow in the node to specify the left-right axis [33, 34]. For example, mice mutant for *left-right dynein* (*Lrd*^*iv*^), have immotile nodal cilia, are unable to generate nodal flow and exhibit laterality defects [35–37]. Reversal of flow by mechanical intervention can invert the left-right axis in wild type embryos and artificial generation of leftward flow can rescue situs defects in *Lrd*^*iv*^ mice [35].

Cilia exist as one of two main types: motile cilia or primary/immotile cilia. Primary cilia play essential roles in receiving and transducing signals from the extracellular environment to the cell body and disruption of primary cilia affects multiple signaling pathways [38, 39]. Both motile and sensory/primary cilia have a basic structure of nine peripheral microtubule doublets arranged around the axoneme periphery [38, 40, 41]. While most motile cilia also have a central pair of microtubule doublets (9+2), motile cilia in the node lack the central pair (9+0) allowing generation of rotational rather than planar movement of 9+2 cilia [40]. Inner and outer dynein arms (IDA and ODA, respectively) connect peripheral doublets and function as force generators to drive beating movements of the cilia. Radial spokes radiate from the center of the axoneme and nexin links connect peripheral doublets, providing the structural support necessary for coordinated beating of the cilia. These ciliary components are preassembled within the cytoplasm by distinct assembly complexes and transported along the axoneme by intraflagellar transport (IFT) [41]. For example, nexin-dynein regulatory complexes (N-DRC) attach IDAs and nexin links to microtubules [41]. Disruption of N-DRC complexes does not affect ODAs but result in disorganization of microtubule doublets and abnormal beating of motile cilia without affecting the signaling functions of primary cilia [40]. In contrast to motile cilia, primary cilia do not contain IDAs, ODAs or nexin links and are generally unaffected by disturbance of N-DRC assembly [42]. Mutation of molecules involved in IFT affects transport of structural components of the cilia resulting in malformed, nonfunctional and/or absent motile and primary cilia and abnormal signal transduction [43].

Of the multiple signaling pathways that require cilia for transduction, Sonic hedgehog (Shh) is best characterized [39, 44–47]. Since Shh is required for expression of *Lefty1* in the midline, in many mouse mutants with defective cilia not only is fluid flow in the node affected but also the midline barrier is dysfunctional, resulting in a high proportion of mutant embryos exhibiting left isomerism [46, 48–50]. This is in contrast to the randomization of left isomerism, situs inversus and normal situs observed in mutants with immotile cilia [32, 51]. Because of their essential role in signal transduction in multiple pathways, disruption of genes involved in formation of primary cilia also cause a spectrum of developmental malformations, respiratory, fertility and situs defects, cystic kidneys, obesity and intellectual disability [52]. In contrast, phenotypes associated with disruption of cilia motility result in PCD and are largely limited to respiratory, fertility and situs defects [40].

Our previous studies demonstrated that the *Coiled-coil domain containing-40 (Ccdc40)* gene plays an evolutionarily conserved role in assembly of motile cilia and establishment of the left-right axis [53]. The *Ccdc40*^*lnks*^ mutant mouse line harbors an ENU-induced nonsense mutation truncating the 1,192 amino acid CCDC40 protein at amino acid 792 disrupting a highly conserved coiled-coil domain. A similarly localized nonsense mutation was identified in zebrafish *lok* (*locke*) mutants where pronephron cilia beat abnormally [53, 54]. Comparable altered beating pattern of cilia was also observed in nasal brush biopsies from human patients with null mutations in *CCDC40* [53]. Analysis of axonemal structure in zebrafish and human cells revealed disorganized microtubule doublets and defective assembly of IDAs and nexin links [53]. Additional analysis of cilia in nasal brush biopsies implicates disruption of N-DRC assembly complexes in these defects [53]. These findings along with the lack of developmental malformations associated with loss of signaling functions of primary cilia in both the mouse mutant and humans with null mutation in *CCDC40* suggest that CCDC40 is required for preassembly of N-DRC complexes in motile cilia but not cilia formation itself [53].

Here we present evidence suggesting that *Ccdc40*^*lnks*^ mutants with defects in motile cilia develop left isomerism by a distinct mechanism from mutants with defective IFT. When cilia generated flow is defective, *Cerl2* fails to downregulate and Nodal activity is antagonized equally on both sides. This effectively reduces activation of this pathway resulting in reduced and delayed activation of Nodal and its antagonists in the LPM. This model is supported by the failure to establish *Nodal* expression in the left-LPM in *Ccdc40*^*lnks/lnks*^;*Nodal*^*LacZ/+*^ mutants resulting in a predominance of right not left isomerism. Together these results suggest a model where flow generated by motile cilia is required to ensure robust Nodal pathway activation on the left side of the node initiating the robust and timely left-sided developmental program in the LPM.

## Materials and methods

### Analysis of mutant mouse phenotype

All animal work was conducted according to protocols approved by the Children’s National Medical Center IACUC protocol #000-30-300. The *Ccdc40*^*lnks*^ mouse line and *Nodal*^*tm1Robv*^ (*Nodal*^*LacZ*^) are described previously [10, 53]. Whole-mount in situs were performed as described [55, 56] using the following probes: *Nodal*, *Cerl2* [57], *Lefty2* [58], Sonic hedgehog (*Shh*) [59], *Brachyury* (*T*) [60] and *Lefty1* was synthesized from Image clone 3985141. β-galactosidase staining and fluorescent immunocytochemistry were preformed as described [61, 62] using the indicated antibodies obtained from the Developmental Studies Hybridoma Bank. Statistical significance was determined by the Chi^2^ test (http://www.physics.csbsju.edu).

## Results

### *Ccdc40*^*lnks/lnks*^ mutant embryos show delayed and reduced expression of L-LPM markers

**Fig 1:**
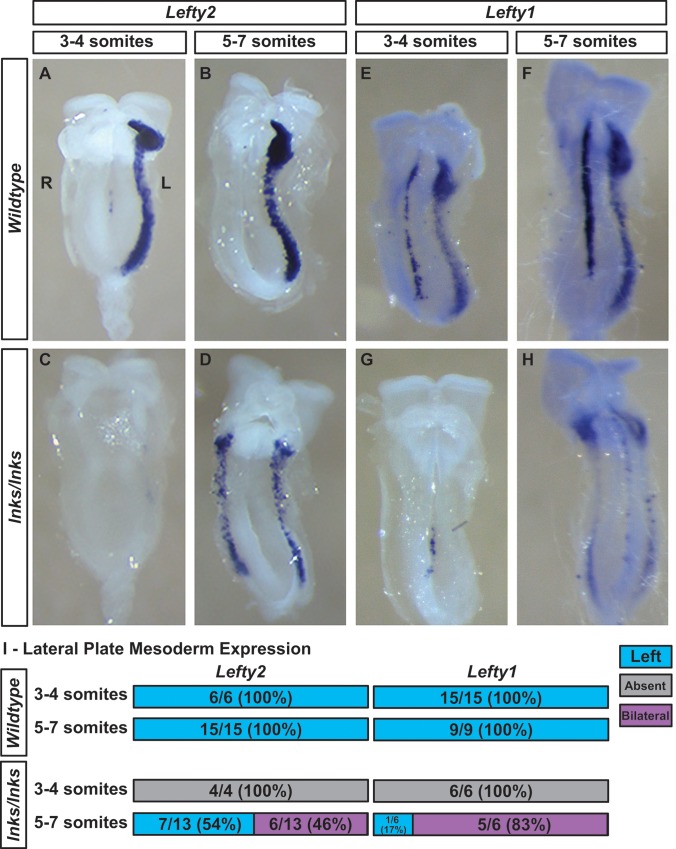
*Ccdc40*^*lnks/lnks*^ embryos show delayed then randomized expression of *Lefty2 and Lefty1*. *Lefty2* (A-D) and *Lefty1* (E-H) expression in wild type (A, B, E, F) and *Ccdc40*^*lnks/lnks*^ (C, D, G, H) embryos at the 3- to 4- (A, C, E, G) and 5- to 7- (B, D, F, H) somite stages. *Lefty1 and Lefty2* is expressed in the L-LPM in wild type embryos at both stages and *Lefty1* is also expressed in the midline. In *Ccdc40*^*lnks/lnks*^ embryos, expression of *Lefty1 and Lefty2* is not detected in the LPM at the 3- to 4-somite stages and expression is randomized between left-sided and bilateral at 5- to 7-somite stages. All embryos with bilateral expression of *Lefty1* and *Lefty2* showed weaker expression in the LPM. Expression of *Lefty1* is weak in the midline of mutants at the 3- to 4-somite stage and mutants with bilateral *Lefty1* expression show reduced expression in the midline. (I.) Quantitation of the number and percentage of embryos with absent (grey), left-sided (blue) and bilateral (magenta) *Lefty1* or *Lefty2* expression in the LPM. Left (L) and Right sides are labeled in panel A.

Almost one-third of *Ccdc40*^*lnks/lnks*^ embryos displayed laterality defects as assessed by lung lobation patterns that include situs inversus (9%; 14/151) and left (21%; 32/151) but not right isomerism [53]. During specification of the left-right axis, *Ccdc40* is expressed in the ciliated nodal pit cells but not perinodal crown cells in addition to the midline [53]. To understand the developmental mechanisms leading to laterality phenotypes in *Ccdc40*^*lnks/lnks*^ mutants with defective motile cilia, the expression of LPM markers were examined. As previously demonstrated [29], *Lefty2* is transiently expressed in the L-LPM in 100% of wild type embryos between the 3- and 7-somite stages (Fig 1A, 1B and 1I). By contrast, *Lefty2* expression was either absent (4/17; 24%) or bilaterally expressed (6/17; 35%) in the L-LPM in over half of *Ccdc40*^*lnks/lnks*^ embryos (Fig 1C, 1D and 1I). Interestingly, when broken down by somite stage, all of the mutants with absent expression of *Lefty2* were between 3- to 4-somite stages (wild type compared to mutant: Chi^2^ = 11.0, df = 1, p = 0.001). By the 5- to 7-somite stages, *Lefty2* expression in *Ccdc40*^*lnks*^ mutants was randomized between left-sided and bilateral (Chi^2^ = 8.81, df = 1, p = 0.003). While the intensity of *Lefty2* expression in mutants with normal situs was comparable to wild type littermates (not shown), expression levels in mutants with bilateral expression were consistently reduced (Fig 1B versus 1D).

Similar results were observed with expression of *Lefty1*. 100% (n = 24) of wild type embryos between the 3- and 7-somite stages showed left-sided expression of *Lefty1* (Fig 1E, 1F and 1I), while *Lefty1* was detected in the L-LPM in only 8% (1/12) of *Ccdc40*^*lnks/lnks*^ embryos, 42% (5/12) showed bilateral expression and 50% (6/12) of mutant embryos showed no detectable expression in the LPM (Fig 1G, 1H and 1I). As with *Lefty2*, all mutants with absent *Lefty1* expression in the LPM were between 3- and 4-somites stages (Chi^2^ = 21.0, df = 1, p = 0.000) and by 5- to 7-somite stages, expression was randomized (Chi^2^ = 11.2, df = 1, p = 0.001). As with *Lefty2*, expression of *Lefty1* in the LPM and midline was reduced in mutants with left isomerism (Fig 1H versus 1F). These results suggest a delayed and reduced initiation of midline and LPM *Lefty1/2* expression in *Ccdc40*^*lnks*^ mutants.

**Fig 2:**
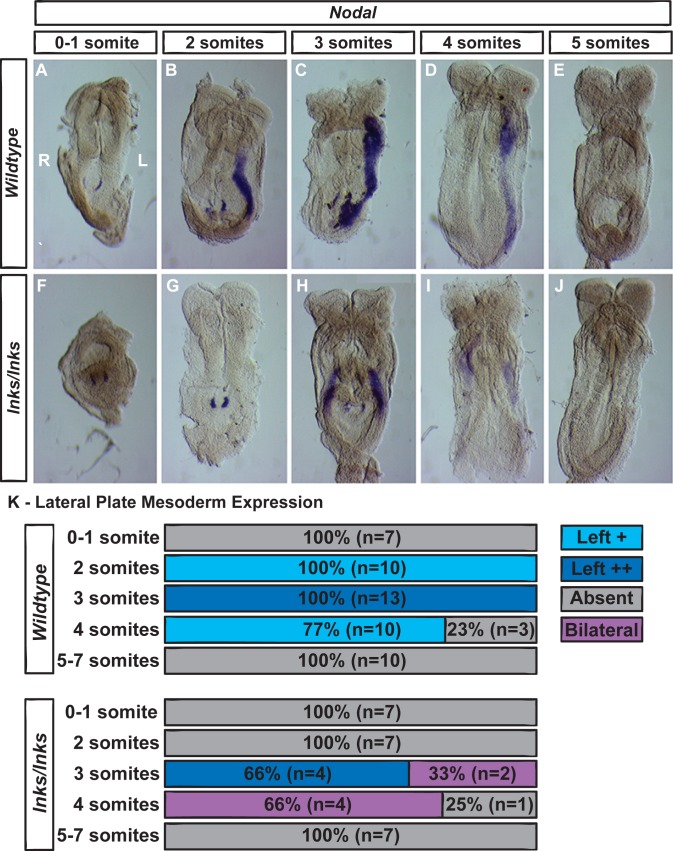
LPM expression of *Nodal* is delayed and then randomized in *Ccdc40*^*lnks/lnks*^ embryos. *Nodal* expression in wild type (A-E) and *Ccdc40*^*lnks/lnks*^ (F-J) embryos at the indicated somite stages. Expression of *Nodal* in the LPM begins at the 2-somite stage in wild type embryos, increases in 3-somite stage embryos and becomes reduced in 4-somite stage followed by extinction in 5- to 7-somite stage embryos. In *Ccdc40*^*lnks/lnks*^ mutant embryos, expression is not detected at the 2-somite stage. Bilateral and weak expression is observed in some embryos at the 3-somite stage. (K.) Quantitation of the number and percentage of embryos with absent (grey), weak left-sided (light blue), strong left-sided (dark blue) or weak bilateral (magenta) *Nodal* expression in the LPM. Left (L) and Right sides are labeled in panel A.

Expression of *Lefty2* and *Lefty1* in the LPM is induced by Nodal [9, 22, 24, 25, 27]. To determine if *Nodal* expression in the LPM was similarly reduced and delayed, expression was examined in 0- to 7-somite stage embryos (Fig 2). As previously reported, *Nodal* expression in the LPM of wild type embryos is transiently increased by the 2-somite stage then extinguished by the 5- to 7-somite stages (Fig 2A–2E and 2K and [10, 32]. Expression in the LPM of wild type embryos is weak at the 2-somite stage and localized posteriorly (Fig 2B). The intensity of *Nodal* expression increases in 3-somite stage embryos where it becomes expressed throughout the LPM (Fig 2C and 2D). In 4-somite stage embryos, expression becomes reduced and by 5-somite stages, staining is absent (Fig 2D and 2E). In contrast, none of the *Ccdc40*^*lnks/lnks*^ embryos examined showed LPM expression at the 2-somite stage (n = 7, Fig 2G and 2K; Chi^2^ = 17.0, df = 1, p = 0.000). 3-somite stage *Ccdc40*^*lnks/lnks*^ embryos show either robust expression in the L-PM (not shown) or weak bilateral posteriorly localized expression (Fig 2H and 2K; Chi^2^ = 4.84, df = 1, p = 0.028). 4-somite stage *Ccdc40*^*lnks/lnks*^ embryos show primarily weak bilateral expression in the anterior LPM (Fig 2I and 2K; Chi^2^ = 14.3, df = 2, p = 0.001). Together these results indicate that induction of LPM gene expression is delayed in *Ccdc40*^*lnks/lnks*^ embryos and once established, expression is randomized between robust expression in the L-LPM or weak bilaterally expression.

### *Lefty1* expression in the midline is reduced in *Ccdc40*^*lnks/lnks*^ embryos

**Fig 3:**
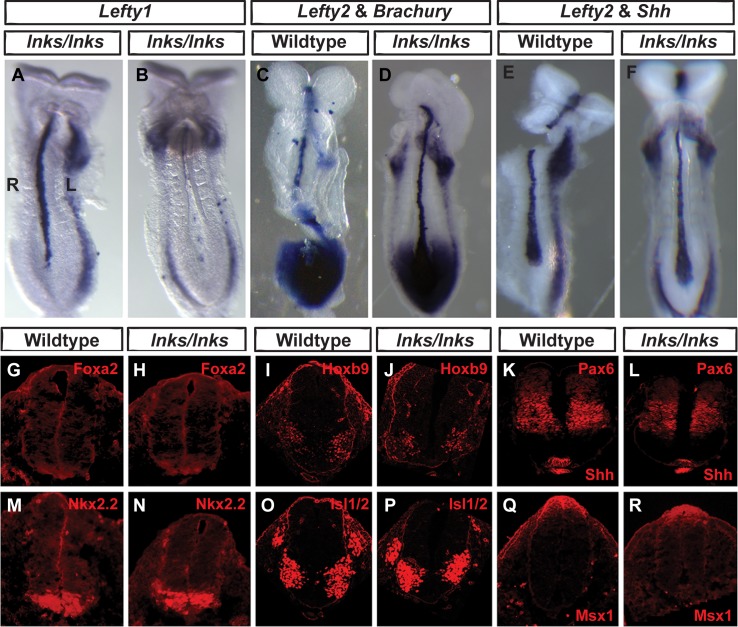
Midline signaling in *Ccdc40*^*lnks/lnks*^ embryos. A, B. Expression of *Lefty1* in the midline is reduced in *Ccdc40*^*lnks/lnks*^ embryos with bilateral (B) compared to L-LPM (A) expression of *Lefty1*. C-F. Double *in situ* hybridizations showing normal expression levels of *Lefty2* (LPM) with either *Brachyury* (C, D) or *Shh* (E, F) in the midline of wild type (C, E) or *Ccdc40*^*lnks/lnks*^ mutant embryos with left isomerism (D, F). G-R. Fluorescent immunocytochemistry demonstrating similar dorsal-ventral distribution of the indicated antigens in the spinal neural tube of wild type (G, I, K, M, O, Q) and *Ccdc40*^*lnks/lnks*^ mutants (H, J, L, N, P, R). Data are representative of 2–3 embryos per genotype. Left (L) and Right sides are labeled in panel A.

In addition to its expression in the L-LPM, *Lefty1* is expressed in the floorplate providing a barrier to prevent Nodal signaling from crossing the midline [29]. Interestingly, all *Ccdc40*^*lnks/lnks*^ embryos identified with bilateral *Lefty1* expression (n = 5) exhibited either absent *Lefty1* expression in the midline or reduced expression compared to wild type littermates (Fig 1H and 1I and Fig 3B). In contrast, the single *Ccdc40*^*lnks/lnks*^ mutant identified with asymmetrical expression of *Lefty1* in the L-LPM showed robust expression in the midline (Fig 3A). Yet, the midline is specified in *Ccdc40*^*lnks/lnks*^ embryos evident by the expression of *Brachyury* in mutant embryos with bilateral *Lefty2* expression (Fig 3C and 3D).

*Lefty1* expression in the mouse midline requires Shh signaling [63]. Primary cilia are required for signal transduction of multiple ligands including Shh signaling [38, 39, 44–47]. While the preponderance of data suggest mutation of *CCDC40* affects motile but not primary cilia and *Ccdc40*^*lnks/lnks*^ embryos do not exhibit other hallmarks of Shh dysfunction including polydactyl, neural tube defect or other gross morphological defects, abnormalities in the morphology of nodal cilia were observed in *Ccdc40*^*lnks/lnks*^ mutants and *Ccdc40* is expressed in the node and midline during specification of the left-right axis [53]. Thus to rule out an effect on Shh signal transduction in primary cilia we examined dorsal-ventral patterning of the spinal cord in *Ccdc40*^*lnks/lnks*^ mutants, a sensitive readout of Shh signaling [64, 65]. Robust expression of *Shh* was detected in the midline of *Ccdc40*^*lnks/lnks*^ embryos with bilateral expression of *Lefty2* (Fig 3E and 3F). Furthermore, expression of a panel of dorsal-ventral patterning markers were unaltered in the spinal cord of *Ccdc40*^*lnks/lnks*^ mutants (Fig 3G–3R). These results confirm that *Ccdc40* is required for cilia motility but not the signaling functions of primary cilia, at least with respect to Shh signaling in the midline during dorsal-ventral patterning of the spinal cord. Thus, reduced *Lefty1* expression in the midline is not likely due to decreased Shh signaling.

### Randomized perinodal expression of *Cerl2* and *Nodal* in *Ccdc40*^*lnks/lnks*^ embryos

**Fig 4:**
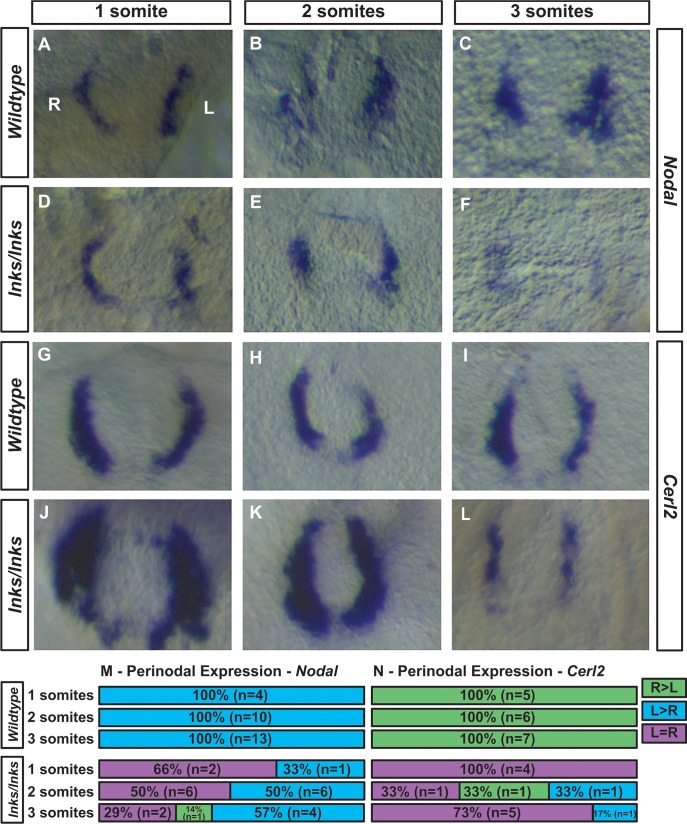
Asymmetric perinodal expression of *Cerl2* and *Nodal* is delayed and then randomized in *Ccdc40*^*lnks/lnks*^ embryos. *Nodal* (A-F) and *Cerl2* (G-L) expression around the node of wild type (A-C, G-I) or *Ccdc40*^*lnks/lnks*^ embryos (D-F, J-L) at the indicated somite stages. M, N. Quantitation of the number and percentage of embryos with right > left (green), left > right (blue) or unbiased (left = right, L = R, magenta) perinodal *Nodal* (Q) or *Cerl2* (R) expression. Left (L) and Right sides are labeled in panel A.

Since *Nodal* expression in the LPM is downstream of initial asymmetries in perinodal *Nodal* expression [9, 22–24], we examined expression of *Nodal* around the node. As previously described [10, 11, 14, 32, 66], higher levels of perinodal *Nodal* expression are found on the left side of the node in 1- to 3-somite stage wild type embryos (n = 27; Fig 4A–4C and 4M). In contrast, biased left-sided expression of perinodal *Nodal* was found in only half of *Ccdc40*^*lnks/lnks*^ mutants examined (11/22; Fig 4D–4F and 4M; Chi^2^ = 17.4, df = 2, p = 0.000). Biased *Nodal* expression in the perinodal region is initiated by downregulation of the Nodal antagonist *Cerl2* on the left side and requires cilia generated fluid flow in the node [11, 13, 22, 67]. In 1-somite stage wild type embryos, *Cerl2* expression is asymmetric with higher levels on the right side of the node (n = 5; Fig 4B and 4N). Biased expression continues through the 3-somite stage (n = 18; Fig 4H, 4I and 4N). In contrast, expression of *Cerl2* in *Ccdc40*^*lnks/lnks*^ embryos remains symmetrical at the 1-somite stage (n = 4; Fig 4J and 4N; Chi^2^ = 9.0, df = 1, p = 0.003) and then becomes randomized in 2–3 somite stage mutants (n = 9; Fig 4K and 4L; Chi^2^ = 18.2, df = 2, p = 0.000). These results indicate that laterality defects in *Ccdc40*^*lnks*^ mutants originate from delayed and then randomized *Cerl2* expression, consistent with defective fluid flow in the node of in *Ccdc40*^*lnks*^ mutants.

### *Ccdc40*^*lnks/lnks*^ interacts with *Nodal*^*LacZ/+*^

**Fig 5:**
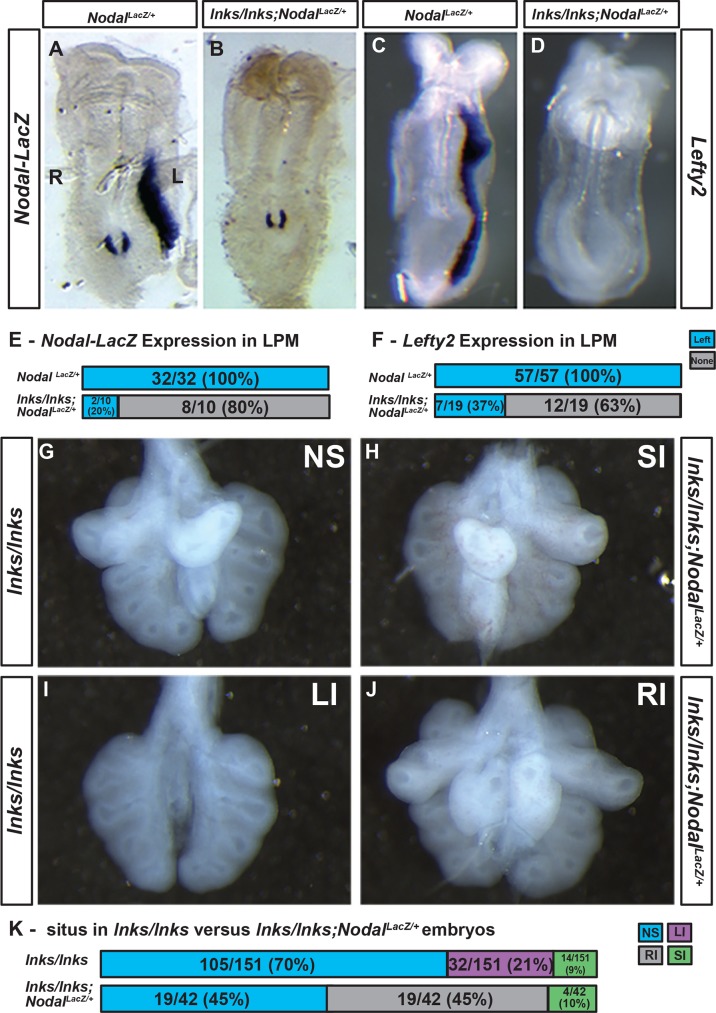
Modification of the *Ccdc40* mutant laterality phenotype with reduced *Nodal* gene dosage. A, B. Beta-galactosidase activity in E8.5 *Nodal*^*LacZ/+*^ (A) or *Ccdc40*^*lnks/lnks*^*;Nodal*^*LacZ/+*^ (B) mutant embryos. Wild type embryos (A) show staining in the left (L) but not right (R) LPM indicating normal situs. (B) Beta-galactosidase staining in the LPM of the majority of *Ccdc40*^*lnks/lnks*^*;Nodal*^*LacZ/+*^ mutant embryos was undetectable even with intense perinodal staining. C, D. *Lefty2* is expressed in the L-LPM of E8.5 *Ccdc40*^*+/+*^*;Nodal*^*LacZ/+*^ embryos but the majority of *Ccdc40*^*lnks/lnks*^*;Nodal*^*LacZ/+*^ mutant embryos (D) fail to express *Lefty2* in the LPM. E, F. Quantitation of *Nodal-LacZ* (E) and *Lefty2* (F) expression in LPM of wild type and *Ccdc40*^*lnks/lnks*^*;Nodal*^*LacZ/+*^ mutant embryos. Normal left-sided expression (Blue) and absent expression (Grey) in the LPM. G-J. Lungs dissected from E11.5 *Ccdc40*^*lnks*^ mutant embryos. G. Normal situs in a *Ccdc40*^*lnks/lnks*^ mutant with a single lobe of the left lung and four lobes of the right. I. Left isomerism in a *Ccdc40*^*lnks/lnks*^ mutant showing bilateral single lobed lungs. The majority of *Ccdc40*^*lnks/lnks*^*;Nodal*^*LacZ/+*^ mutant lungs showed either normal situs (not shown) or right isomerism (J). A small percentage of *Ccdc40*^*lnks/lnks*^ (not shown) and *Ccdc40*^*lnks/lnks*^*;Nodal*^*LacZ/+*^ (H) mutants showed situs inversus. K. Quantitation of the number and percentage of lungs showing normal situs (NS, blue), left isomerism (LI, magenta), right isomerism (RI, grey) or situs inversus (SI, green) in E11.5–15.5 *Ccdc40*^*lnks/lnks*^ and *Ccdc40*^*lnks/lnks*^*;Nodal*^*LacZ/+*^ mutants.

Our data demonstrate that mutant embryos with left-isomerism show reduced expression of *Nodal* and Nodal-induced transcripts in the LPM and midline. Failure to bias *Nodal* and *Cerl2* expression around the node could have the effect of reducing the robustness of the Nodal signal transmitted to the LPM. If Nodal activation were suboptimal in *Ccdc40*^*lnks/lnks*^ embryos, further reducing Nodal activity would compound this deficit resulting in inadequate Nodal expression in the L-LPM to initiate the left-sided developmental program. To test this model we crossed *Ccdc40*^*lnks/+*^ with the *Nodal*^*LacZ*^ mutant mouse where the Nodal gene is disrupted by insertion of a LacZ cassette [10]. In *Nodal*^*LacZ/+*^ embryos, Beta-galactosidase (β-gal) activity is detected in the L-LPM (Fig 5A and 5E). In contrast, β-gal activity was detected on the left side in only one-fifth of *Ccdc40*^*lnks/lnks*^*;Nodal*^*LacZ/+*^ embryos (2/10) and the remainder showed no staining in the LPM (Fig 5B and 5E; Chi^2^ = 31.6, df = 1, p = 0.000). Consistent with our hypothesis, bilateral staining was not detected in any of the *Ccdc40*^*lnks/lnks*^*;Nodal*^*LacZ/+*^ embryos examined. Similar to *Nodal-LacZ* expression, one-third of *Ccdc40*^*lnks/lnks*^*;Nodal*^*LacZ/+*^ embryos exhibited left-sided expression of *Lefty2* (7/19) and the remainder did not express *Lefty2* in either the L- or R-LPM (Fig 5C, 5D and 5F; Chi^2^ = 42.8, df = 1, p = 0.000).

Our analysis of *Nodal-lacZ* and *Lefty2* expression indicates that the majority of *Ccdc40*^*lnks/lnks*^*;Nodal*^*LacZ/+*^ mutant embryos would exhibit right isomerism instead of the left isomerism observed in *Ccdc40*^*lnks/lnks*^ mutants. To test if this were the case, we examined the morphology of lungs from *Ccdc40*^*lnks/lnks*^*;Nodal*^*LacZ/+*^ mutants at later stages of development when lung lobation patterns could be assessed. The majority of *Ccdc40*^*lnks/lnks*^ mutant lungs showed normal situs with a single lobe of the left lung and four lobes on the right (105/151; 70%; Fig 5G and 5K). One quarter of the mutant lungs showed left isomerism (32/151; 21%) and a small percentage situs inversus (14/151; 9%; Fig 5I and 5K and not shown). In contrast, almost half of the *Ccdc40*^*lnks/lnks*^*;Nodal*^*LacZ/+*^ mutant lungs examined exhibited right isomerism (19/45; 45%), a small percentage situs inversus (4/45; 10%) and the remaining normal situs (19/45; 45%; Fig 5H–5K; Chi^2^ = 80.2, df = 3, p = 0.000). These results indicate that in many embryos, reduction of *Nodal* gene dosage reduces pathway activation below the threshold required to activate the left-sided developmental program.

## Discussion

**Fig 6:**
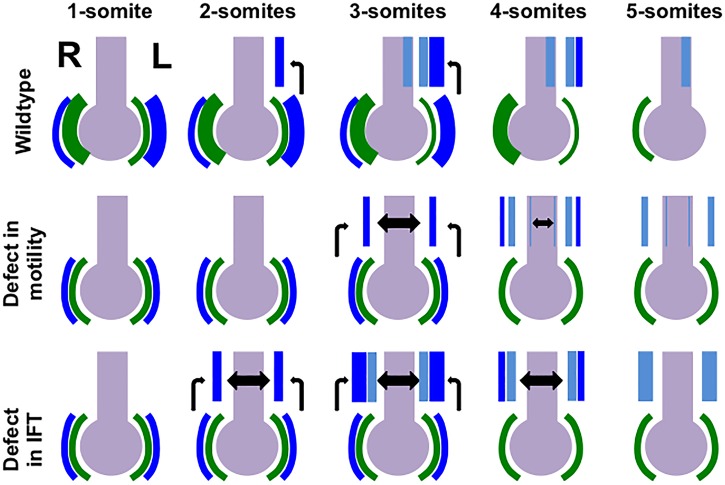
Model for Generation of Left Isomerism in *Ccdc40* versus Shh pathway mutants. In 1-somite stage wild type embryos, *Cerl2* expression (green) becomes asymmetric with reduced expression on the left side of the node in response to fluid flow. With reduced expression of its antagonist on the left, Nodal activity and expression (dark blue) increases on the left side and decreases on the right. In 2-somite stage wild type embryos, Nodal induce *Nodal* expression in the L-LPM and by the 3-somite stage robust expression of *Nodal* along with *Lefty1* and *Lefty2* (light blue) is detected in the L-LPM and *Lefty1* in the midline (light blue). In 4-somite stage embryos *Nodal* expression is reduced but *Lefty1* and *Lefty2* are strongly expressed in the L-LPM. *Lefty1* expression in the midline and *Cerl2* around the node inhibits Nodal signaling in the R-LPM. With lack of nodal flow in *Ccdc40*^*lnks*^ and other cilia mutants with intact Shh signaling, *Cerl2* and *Nodal* expression remains symmetric. Continued *Cerl2* mediated antagonism of Nodal results in reduced Nodal signaling around the node and *Nodal* and *Lefty1/2* expression in the LPM is delayed till the 3- and 4-somite stages, respectively. In a proportion of embryos where symmetry is not broken, Nodal signaling is reduced and bilateral. This is in contrast to IFT mutants with defects in Shh signaling. In these embryos, Nodal signaling is robustly induced bilaterally compounded by failure to activate the midline barrier.

In this study we characterized the developmental mechanisms leading to laterality defects in *Ccdc40*^*lnks*^ mutant embryos. Mutants show delayed induction then randomization of L-LPM markers. Interestingly, in mutant embryos with bilateral expression of L-LPM markers, expression of *Nodal* and its antagonists *Lefty1* and *Lefty*2 are delayed and reduced, as is *Lefty1* expression in the midline. Around the node, biased expression of *Cerl2* and *Nodal* is also delayed then randomized. Our data suggest a model (Fig 6) where if no asymmetry is generated, as when flow is disrupted in *Ccdc40* mutants, robust activation of the Nodal pathway does not occur. With symmetrical expression of *Cerl2*, Nodal activity is reduced on both sides of the node, as is the transfer of Nodal to the LPM. This results in delayed and reduced induction of *Nodal* and *Lefty1/2* expression in the LPM and *Lefty1* in the midline. We tested this model by crossing *Ccdc40*^*lnks*^ with a *Nodal* mutant line, revealing that reduction of *Nodal* gene dosage on a *Ccdc40*^*lnks*^ mutant background results in failure to establish left-sided gene expression and right isomerism.

During establishment of left-right axis in the mouse, *Ccdc40* is expressed exclusively in the nodal pit cells and midline [53]. Though not formally shown in the node, *CCDC40* is required for movement of cilia, suggesting that fluid flow in the node is likely also disrupted in *Ccdc40*^*lnks*^ mutants. Human patients as well as zebrafish mutants and morphants with mutations in *CCDC40* show situs inversus and heterotaxia [53]. Nodal cilia in *Ccdc40* mouse mutants are shorter [53] and pronephric cilia in zebrafish *lok* mutants show motility defects [54]. Importantly, high speed video microscopy analysis of respiratory cilia from nasal brush biopsies obtained from Primary Ciliary Dyskinesia (PCD) patients with *CCDC40* mutations demonstrate abnormal beat patterns and rigid appearance [53]. Further analysis revealed that *CCDC40* is required for assembly of motile cilia [53]. Cilia assembly occurs at the base of the cilium in the cytoplasm with recruitment of protein complexes that preassemble ciliary structures such as IDAs, ODAs, nexin and radial spokes [41]. After pre-assembled in the cytoplasm, these structures are then transported from the basal body to functional assembly sites along the axonome [41]. Ultrastructural analysis reveals multiple defects in both human and zebrafish cilia mutant for *CCDC40* [53]. These include structural defects in the organization of axonemes such as duplicated or misplaced microtubule doublets. While ODAs appeared normal, IDAs are missing or reduced in number and radial spoke and nexin links were affected. In fact, *CCDC40* and the related *CCDC39* account for the vast majority of PCD cases with these characteristic cillary ultrastructural defects [53, 68–70].

How other negative and positive feedback loops that regulate left-right axis formation are altered in *Ccdc40* mutants remains to be determined. Interestingly, *Cerl2* expression was consistently increased in perinodal crown cells in *Ccdc40* mutants. In addition to Nodal, Cerl2 also antagonizes Wnt signaling and Wnt signaling in turn inhibits *Cerl2* expression [12, 14]. Thus it is possible that failure to downregulate *Cerl2* results in increased antagonism of Wnt, further increasing *Cerl2* expression. Dysregulation of Wnt and other positive and negative feedback loops downstream of motile cilia in the node could also contribute to the observed laterality defects.

We found an interesting genetic interaction of *Ccdc40*^*lnks*^ with *Nodal* and similar genetic interactions are reported in other studies [57, 71]. For example, mutation of *left-right dynein* (*Lrd*) in the *Lrd*^*iv*^ line results in defective nodal flow and these mutants show a similar interaction with the Nodal pathway resulting in right instead of left isomerism [36, 37, 49, 51]. Cillary defects in *Lrd*^*iv*^ mice are well characterized, demonstrating immotile cilia in the node that are unable to generate nodal flow resulting in approximately half of *Lrd*^*iv/iv*^ mutants showing situs defects [36, 37, 49]. However, loss of a single copy of a Nodal receptor (ActRIIB) on the *Lrd*^*iv/iv*^ mutant background transforms the predominant phenotype of *Lrd*^*iv/iv*^*;ActRIIB*^*-/+*^ compound mutant embryos to right instead of left isomerism [71]. Similar genetic interactions were found in *Arl13b*^*hnn/hnn*^*;Nodal*^*LacZ/+*^ compound mutants that do not display alterations in Shh signaling during specification of the left-right axis [57]. Yet, it remains unknown if mutation of *Arl13b* results in defects in generation or detection of Nodal flow [57]. Interestingly, transformation of the phenotype with genetic interaction of *Ccdc40*^*lnks*^, *Arl13b*^*hnn*^ or *Lrd*^*iv*^ with the Nodal pathway is fundamentally different than what is seen in cilia mutants affecting the Shh pathway that also develop left isomerism. Bilateral *Nodal* expression is observed in Shh pathway mutants at early somite stages due to failure to induce *Lefty1* in the midline and left isomerism still predominates in these mutants with reduced Nodal gene dosage [44, 46, 57, 63]. While *Ccdc40*^*lnks*^ mutants with left isomerism show reduced *Lefty1* in the midline, *Lefty1* is expressed at low levels in the midline and other midline markers and indicators of Shh signaling appear normal. Thus we suggest that the developmental mechanism leading to left isomerism in *Ccdc40*^*lnks*^, *Arl13b*^*hnn*^ and *Lrd*^*iv*^ mutants is different than that observed with disruption of the Shh pathway (Fig 6). We propose that when cilia generated flow is defective, *Cerl2* is not downregulated and Nodal activity is antagonized equally on both sides. This effectively reduces activation of this pathway resulting in reduced and delayed activation of Nodal and its antagonists in the LPM. Thus cilia function to ensure robust asymmetric Nodal pathway activation needed to initiate a proper and timely developmental program in the L-LPM.
